# miRNA alteration is an important mechanism in sugarcane response to low-temperature environment

**DOI:** 10.1186/s12864-017-4231-3

**Published:** 2017-10-30

**Authors:** Yuting Yang, Xu Zhang, Yachun Su, Jiake Zou, Zhoutao Wang, Liping Xu, Youxiong Que

**Affiliations:** Key Laboratory of Sugarcane Biology and Genetic Breeding, Ministry of Agriculture and Key Laboratory of Crop Genetics and Breeding and Comprehensive Utilization, Ministry of Education, College of Crop Science, Fujian Agriculture and Forestry University, Fuzhou, 350002 China

**Keywords:** *Saccharum* spp., High-throughput sequencing, *miR156*, Cold, Transcriptome

## Abstract

**Background:**

Cold is a major abiotic stress limiting the production of tropical and subtropical crops in new production areas. Sugarcane (*Saccharum* spp.) originates from the tropics but is cultivated primarily in the sub-tropics where it frequently encounters cold stress. Besides regulating plant growth, miRNAs play an important role in environmental adaption.

**Results:**

In this study, a total of 412 sugarcane miRNAs, including 261 known and 151 novel miRNAs, were obtained from 4 small RNA libraries through the Illumina sequencing method. Among them, 62 exhibited significant differential expression under cold stress, with 34 being upregulated and 28 being downregulated. The expression of 13 miRNAs and 12 corresponding targets was validated by RT-qPCR, with the majority being consistent with the sequencing data. GO and KEGG analysis indicated that these miRNAs were involved in stress-related biological pathways. To further investigate the involvement of these miRNAs in tolerance to abiotic stresses, sugarcane *miR156* was selected for functional analysis. RT-qPCR revealed that *miR156* levels increased in sugarcane during cold, salt and drought stress treatments. *Nicotiana benthamiana* plants transiently overexpressing *miR156* exhibited better growth status, lower ROS levels, higher anthocyanin contents as well as the induction of some cold-responsive genes, suggesting its positive role in the plant cold stress response.

**Conclusions:**

This study provides a global view of the association of miRNA expression with the sugarcane response to cold stress. The findings have enriched the present miRNA resource and have made an attempt to verify the involvement of *miR156* in plant response to cold stress.

**Electronic supplementary material:**

The online version of this article (10.1186/s12864-017-4231-3) contains supplementary material, which is available to authorized users.

## Background

Small non-coding RNAs of 18–40 nucleotides (nt) in length, including microRNAs (miRNAs) and small interference RNAs (siRNAs), can regulate gene expression by posttranscriptional mechanisms and epigenetic modifications to influence a number of biological processes [1]. These endogenous 18–25 nt non-coding RNAs [2] can target mRNA cleavage or translational repression by guiding the RNA-induced silencing complex (RISC) to bind to the target mRNA [3]. Primary miRNAs (pri-miRNAs) are transcribed as long precursors in the nucleus, and can be transferred to the cytoplasm to form mature miRNAs after a series of processing steps [4, 5]. The physiological significance of miRNAs in plants has been demonstrated in various biological processes [6, 7]. In order to understand the molecular mechanism of miRNAs in non-model organisms, more miRNAs need to be identified and characterized. With the advantages of identifying large amounts of miRNAs effectively in a short time and at low cost [8], high-throughput sequencing has become the main method for obtaining miRNAs [9, 10].

Cold stress, including chilling (<20 °C) and freezing (< 0 °C), is one important limiting factor that can affect the geographical distribution and planting season of crops, especially for tropical and subtropical crops [11]. Low temperature can suppress plant growth and development by inhibiting metabolic reactions, leading to osmotic, oxidative and other stresses [12]. Plants usually possess a series of strategies to respond to temperature fluctuations, such as the remodeling of cell and tissue structures and gene expression, and metabolism reprogramming [11–13]. Cold-related gene expression can be regulated at the transcriptional, post-transcriptional and post-translational levels [14]. During low temperature stress, C-repeat binding factors (*CBFs*), which bind to dehydration-responsive elements in gene promoters to activate the downstream cold-responsive (*COR*) genes, is regulated by the inducer of C-repeat binding factor expression 1 (*ICE1*). This is known as the ICE-CBF-COR pathway [14, 15]. Furthermore, reports have shown that some miRNAs also take part in the plant response to cold stress and play crucial roles in this process [16, 17]. In sugarcane (*Saccharum* spp.), the up-regulation of *miR319* and down-regulation of its targets, including a *Myb* transcription factor (*GAMyB*) and a *TCP* transcription factor (*PCF5*), were observed in both cold -tolerant and -sensitive sugarcane varieties under treatment at 4 °C [18]. However, their expression changes were delayed in cold tolerant varieties [18]. Similar results were found in *Oryza sativa* L., whereby the overexpression of *osa*-*miR319* led to enhanced cold tolerance in transgenic rice seedlings [19]. In addition, some other cold-related miRNAs, such as *miR156k* from rice [20] and *miR394* from *Arabidopsis* [21], have also been reported.

Sugarcane accounts for ∼80% of the global sugar production and has successfully been developed to be one of the most promising energy biofuels in Brazil [22]. However, sugarcane growth and development is affected by various factors, and due to its tropical origin, domestic varieties are restricted to tropical and subtropical regions [23, 24]. Although low temperature is beneficial for sucrose accumulation in sugarcane [25], it also leads to bud browning, resulting in poor growth [26]. Sugarcane harvest normally begins in winter and continues until spring.

In the present study, a high-throughput small RNA deep sequencing method was used to obtain cold-related miRNAs in sugarcane. The interaction networks of these miRNAs and their targets were investigated based on Gene Ontology (GO) and Kyoto Encyclopedia of Genes and Genomes (KEGG) analysis. In addition, the roles of several miRNAs, such as *miR156, miR168*, *miR169* and *miR408*, in the plant response to abiotic stress, were explored. Our aim was to gain insight into the molecular mechanism of sugarcane cold tolerance and the role of miRNAs in this process.

## Methods

### Samples and small RNA library preparation

The nodes of ROC22 (relative cold sensitivity) and FN39 (relative cold tolerance) sugarcane cultivars (*Saccharum* spp. hybrid) were harvested from the field in the Key Laboratory of Sugarcane Biology and Genetic Breeding, Ministry of Agriculture in Fuzhou City, China. The related information of cold tolerance is indicated in Additional file 10: Fig. S1. These nodes were firstly treated with flowing water for 2 days to promote the sprouting of buds and remove impurities, and then they were cultivated in an incubator (Dongqi, Ningbo, China) at 28 °C in the dark and at 65% relative humidity for 4 days for sprouting using the moisture culture method [26]. The cold treatment (4 °C) was performed on corresponding buds from both cultivars for 0 (before the cold treatment), 3, 12, 24 and 48 h. Three biological replications were used for each time point and 6 buds for each sample. The total RNA of all the collected samples was extracted using Trizol™ Reagent (Invitrogen, Carlsbad, CA), qualified by electrophoresis with a 1% agarose gel and quantified using a NanoVue Plus (GE healthcare, Little Chalfont, UK). Usually, gene expression changes occur early upon cold stress, as attested by already published reports [27, 28] and previous results obtained in our lab (unpublished data). For this reason, samples collected at 0 and 3 h of cold stress treatment were assumed to be suitable for target molecular analyses. Therefore, the total RNA (10 μg) of the 0 and 3 h samples of FN39 and ROC22 cultivars was sent to BGI (Shenzhen, China) on dry ice for high-throughput small RNA deep sequencing using the Illumina HiSeq high-throughput sequencing platform.

Healthy sugarcane plantlets exhibiting constant growth status derived from the tissue culture of the ROC22 cultivar were grown under 16 h light/ 8 h dark conditions at 28 °C for 1 week in a incubator. Then, the plantlets were treated with 250 mM NaCl and 25% PEG8000 as the salt and drought stresses, respectively [29]. The samples subjected to salt and drought treatments for 0, 6, 12 and 24 h were collected for RNA extraction. Three biological replications were setup and 5 plantlets were used for each sample.

### Bioinformatics analysis

Following size fractionation of the 18–30 nucleotide small RNAs and 5′ adaptor ligation and 3′ adaptor ligation, the small RNAs were reversely transcribed into cDNA, amplified and sequenced. The clean reads were obtained from the raw data from small RNA deep sequencing by removing the low-quality reads and impurities, including the reads without insertions; without 3′ -primer; with 5′-primer contaminants; those with poly A tails as well as small fragment sequences. The clean reads were firstly used to analyze the common/specific sequences and length distributions [30]. The ribosomal RNA (rRNA), small cytoplasmic RNA (scRNA), small nucleolar RNA (snoRNA), small nuclear RNA (snRNA) and transfer RNA (tRNA) were annotated and removed from the clean reads via a BLASTn search on the NCBI GenBank database and Rfam (10.1) database (e = 0.01). The remaining clean reads were aligned with the known miRNAs in miRBase (http://www.mirbase.org/) using the procedure and parameter blastall -p blastn -F F -e 0.01. The detail criterion is that firstly align the clean reads to the miRNA precursor in miRBase with no mismatch. Secondly, the obtained tags align with the mature miRNAs in miRBase with at least 16 nt overlap allowing offsets. Those miRNAs satisfying both criteria will be counted to get their expression, and will be analyzed to get base bias on the first position of the identified miRNAs with certain length, and base bias on each position of all identified miRNAs respectively. The novel miRNAs were predicted using the Mireap software (http://sourceforge.net/projects/mireap/) developed by BGI through considering the biological characteristics of miRNA, such as the secondary structure, the location of mature miRNA on its precursor and the minimum free energy [31]. Specific parameters were set as follows: minimal miRNA sequence length was 18; maximal miRNA sequence length was 25; minimal miRNA reference sequence length was 20; maximal miRNA reference sequence length was 23; maximal copy number of miRNAs on reference was 20; maximal free energy allowed for a miRNA precursor was −18 kcal/mol; maximal space between miRNA and miRNA* was 300, minimal base pairs of miRNA and miRNA* was 16; maximal bulge of miRNA and miRNA* was 4; maximal asymmetry of miRNA/miRNA* duplex was 4; flank sequence length of miRNA precursor was 20 [31].

The comparison analysis between the control and the cold-treated sugarcane was performed according to the following steps. The miRNA expression quantity was normalized using the formula: $\mathrm{Normalized}\  \mathrm{expression}={\frac{\mathrm{actual}\  \mathrm{miRNAcount}}{\mathrm{total}\  \mathrm{count}\  \mathrm{of}\  \mathrm{clean}\  \mathrm{reads}}}^{\ast}\mathrm{1,000,000}$. Then, the normalized expression quantity was used to calculate the fold change using the formula:$\mathrm{Fold}\  \mathrm{change}={\mathit{\log}}_2\left(\frac{\mathrm{treatment}}{\mathrm{control}}\right)$.

Mireap software was also used to predict the potential target genes according to the rules as previously reported [32, 33]. In order to identify the functions of potential targets of differentially expressed miRNAs, the number of targets mapping to each gene ontology term in the Gene Ontology database was calculated. Then, the significantly enriched GO terms were obtained using the super geometry inspection method. The GO enrichment analysis can provide clues for the identification of the main biological functions of the potential targets [34]. To further understand the biological functions, KEGG pathway analysis was also conducted, which provides information on the main biochemical pathways and signal transduction pathways the potential targets involved [35]. The GO enrichment analysis was used on predicted target gene candidates of miRNAs. This method firstly map all target gene candidates to GO terms in the database (http://www.geneontology.org/), and calculate gene numbers for each term, and then use hypergeometric test to find significantly enriched GO terms in target gene candidates comparing to the reference gene background. The calculating formula is:$\mathrm{P}=1-\sum \limits_{i=0}^{m-1}\frac{\left(\genfrac{}{}{0pt}{}{M}{i}\right)\left(\genfrac{}{}{0pt}{}{N-M}{n-i}\right)}{\left(\genfrac{}{}{0pt}{}{N}{n}\right)}$, within which N is the number of all genes with GO annotation; n is the number of target gene candidates in N; M is the number of all genes that are annotated to a certain GO term and m is the number of target gene candidates in M. The Bonferroni Correction for the *p*-value is used to obtain the corrected p-value. GO terms with corrected p-value <0.05 are defined as significantly enriched in target gene candidates. KEGG pathway analysis was also used for the target gene candidates. The calculating formula is the same as that in GO analysis. In this formula, N is the number of all genes with KEGG annotation, n is the number of target gene candidates in N, M is the number of all genes annotated to a certain pathway, and m is the number of target gene candidates in M. Genes with FDR(false discovery rate) <0.05 are considered as significantly enriched in target gene candidates. The KEGG analysis reveals the main pathways which the target gene candidates are involved in.

### Expression validation of miRNAs and potential target genes by RT-qPCR

The cold responsive genes, *CBF1* (C-repeat binding factor gene), *CBF3* and *NAC23* (NAM-ATAF-CUC gene), have been reported to be induced by cold stress and were used to verify the efficiency of cold treatment in this study by the reverse transcription quantitative real-time polymerase chain reaction (RT-qPCR) method. In order to validate the presence and expression levels of miRNAs, 13 miRNAs were randomly detected by RT-qPCR method. Total RNA was extracted from the samples of 0, 3, 12, 24 and 48 h cold-treated sugarcane buds using Trizol™ Reagent (Invitrogen, CA), followed by DNase (Promega, USA) treatment to remove the genomic DNA. For the miRNA, the cDNA synthesis was performed according to the instruction of the TaqMan MicroRNA Reverse Transcription Kit (Applied Biosystems, USA). For each miRNA, the specific stem loop primer (Additional file 1: Table S1) was added to the reaction system. The reaction was performed at 16 °C for 30 min, 42 °C for 30 min and 85 °C for 5 s. All cDNA samples were 25-fold diluted and then used as a template in the miRNA RT-qPCR analysis. For the potential target genes, the cDNA synthesis was performed according to the instruction of the PrimeScript™ RT Reagent Kit (TaKaRa, Dalian, China). The reaction procedure included reverse transcription at 37°C for 15 min and the inactivation of reverse transcriptase with heat treatment at 85 °C for 5 s. The expression patterns of miRNAs and potential targets were detected according to the SYBR Green Master (ROX) (Roche, China) manufacturer instruction on a 7500 real time PCR system (Applied Biosystems, USA) (Additional file 2: Table S2). 18S ribosomal RNA (*18S* rRNA) [26] and glyceraldehyde-3-phosphate dehydrogenase (*GAPDH*) [18] were used as the internal control for miRNA and potential targets, respectively. The expression was calculated using the 2^-△△Ct^ method [36].

All the statistical analysis was conducted using the Statistical Product and Service Solutions (SPSS20.0 version) software. Data was expressed as the mean ± standard error (SE). Significance (*p*-value <0.05) was calculated using one-way Analysis of Variance (ANOVA) followed by Duncan’s multiple range test.

### The transient expression of miRNA in *Nicotiana benthamiana*

The precursor of *miRNA*, *MIRNA*, cloned from sugarcane was sub-cloned with *Bam*H I and *Xba* I sites into the overexpression vector pCAMBIA 1301, and then transferred into the *Agrobacterium tumefaciens* strain EHA105. After inoculation in LB medium containing kanamycin (50 μg/mL) and rifampicin (34 μg/mL) twice, the cells of the EHA105 strain were collected and diluted to OD_600_ = 0.8 with Murashige and Skoog (MS) liquid medium (containing 200 mM acetosyringone). The *Agrobacterium*-mediated transformation was performed according to a previously described method [37]. The 1 mL syringe was used to infiltrate diluted bacterial suspension into the leaves of tobacco plants at the 6–8-leaf stage. After cultivation for 48 h under 16 h light/ 8 h dark at 23 °C conditions, the injected tobacco plants were treated with 4 °C stress for 12, 24and 48 h in an incubator (Dongqi, Ningbo, China). Then the injected tobacco leaves were collected for RNA extraction and DAB staining. Six biological replications were used.

3, 3′-diaminobenzidine (DAB) solution staining was used to assess H_2_O_2_ production [37]. The collected leaves were put into DAB solution (1 mg/mL, pH = 5.8) overnight under dark condition for staining. After boiling in 95% alcohol until the green color faded, the leaves were rinsed in 95% alcohol and photographed.

Anthocyanin quantification was performed according to a previously described method [38]. Briefly, 0.2 g fresh injected tobacco leaves were exacted with MeOH: HCl: H_2_O (80:5:15) at 4 °C overnight in the dark. After centrifugation at 15,000×*g* for 5 min, the anthocyanin levels in the extracts were qualified at 530 nm (T6 spectrophotometer, Beijing Purkinje General Instruments).

## Results

### Data analysis of small RNA libraries

**Fig. 1:**
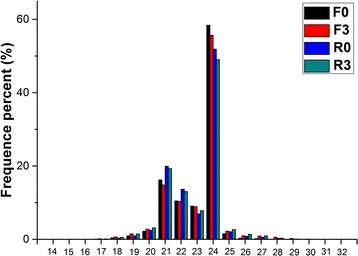
The length distribution of small RNAs in 4 small RNA libraries. F0 and F3 represent the bud samples from sugarcane cultivar FN39 with 0 and 3 h cold (4 °C) treatment, respectively, while R0 and R3 represent the similar samples from cultivar ROC22 with 0 and 3 h cold (4 °C) treatment, respectively

In order to investigate the expression of miRNAs in sugarcane under cold stress, samples were collected at 0, 3, 12, 24 and 48 h over the 4 °C treatment. To validate the cold treatment, the expression of three cold responsive genes (*CBF1*, *CBF3* and *NAC23*) was examined in these collected samples by RT-qPCR. The results showed that the transcripts of the three genes were all increased compared to those under control condition (Additional file 11: Figure S2), indicating that the cold treatment to sugarcane was effective in this study. In order to identify the miRNAs and other small RNAs involved in cold tolerance, two sugarcane cultivars including FN39 (relative cold tolerance) and ROC22 (relative cold sensitivity) were used to generate four small RNA libraries: F0 (samples of FN39 cultivar without cold treatment), F3 (samples of FN39 cultivar with 3 h cold treatment), R0 (samples of ROC22 cultivar without cold treatment) and R3 (samples of ROC22 cultivar with 3 h cold treatment). These libraries were sequenced by Illumina sequencing, which generated between 20 and 25 million clean reads after filtering out the low quality reads and contaminants, which accounted for 0.78% (F0), 0.97% (F3), 0.78% (R0) and 0.90% (R3) of the raw reads, respectively (Table 1). In all the above libraries, the reads with 24 nt and 21 nt lengths were the most abundant (Fig. 1). The analysis of 18–30 nt miRNA nucleotide bias showed that the first base of 21 nt and 22 nt miRNAs was uracil (U), and the miRNA nucleotide bias at the tenth-eleventh position, usually as the cleavage site, was an adenine (A) in all these libraries.

**Table 1:** Small RNA categorization in sugarcane

Categorization	Unique sRNA (%)				Total sRNA (%)			
	F0 (%)	F3 (%)	R0 (%)	R3 (%)	F0 (%)	F3 (%)	R0 (%)	R3 (%)
miRNA	67,373 (0.53)	57,249 (0.56)	59,960 (0.54)	58,772 (0.58)	2,386,352 (9.41)	1,638,016 (7.89)	2,635,873 (10.12)	2,302,177 (9.52)
rRNA	56,395 (0.44)	80,912 (0.79)	81,039 (0.73)	98,210 (0.97)	729,453 (2.88)	1,613,788 (7.78)	1,453,854 (5.58)	2,451,555 (10.14)
repeat	412 (0)	345 (0)	447 (0)	376 (0)	957 (0)	799 (0)	1019 (0)	774 (0)
snRNA	4811 (0.04)	4600 (0.05)	4096 (0.04)	4259 (0.04)	12,452 (0.05)	12,260 (0.06)	12,838 (0.05)	13,164 (0.05)
snoRNA	2152 (0.02)	2197 (0.02)	2584 (0.02)	2396 (0.02)	4567 (0.02)	4583 (0.02)	5840 (0.02)	5522 (0.02)
srpRNA	0 (0)	2 (0)	0 (0)	0 (0)	0 (0)	2 (0)	0 (0)	0 (0)
tRNA	9007 (0.07)	13,069 (0.13)	13,810 (0.12)	15,819 (0.16)	214,125 (0.84)	17,084,377 (1.94)	467,304 (1.79)	696,897 (2.88)
unann	12,602,337 (98.8)	10,044,731 (98.45)	10,917,735 (98.54)	9,907,437 (98.22)	22,010,746 (86.8)	17,084,337 (1.94)	21,461,967 (82.42)	18,702,763 (77.37)
Clean reads	12,742,487 (100)	10,203,105 (100)	11,079,671 (100)	10,087,269 (100)	25,358,652 (100)	20,755,724 (100)	26,038,695 (100)	24,172,852 (100)
Raw reads	–	–	–	–	25,556,975	20,959,989	26,243,698	24,392,550

The number represents the raw data generated directly from small RNA deep sequencing. F0 and F3 represent the bud sample from sugarcane cultivar FN39 with 0 h and 3 h cold (4 °C) treatment, respectively, while R0 and R3 represent the similar samples from cultivar ROC22, respectively

### Identification of known miRNAs in sugarcane

**Fig. 2:**
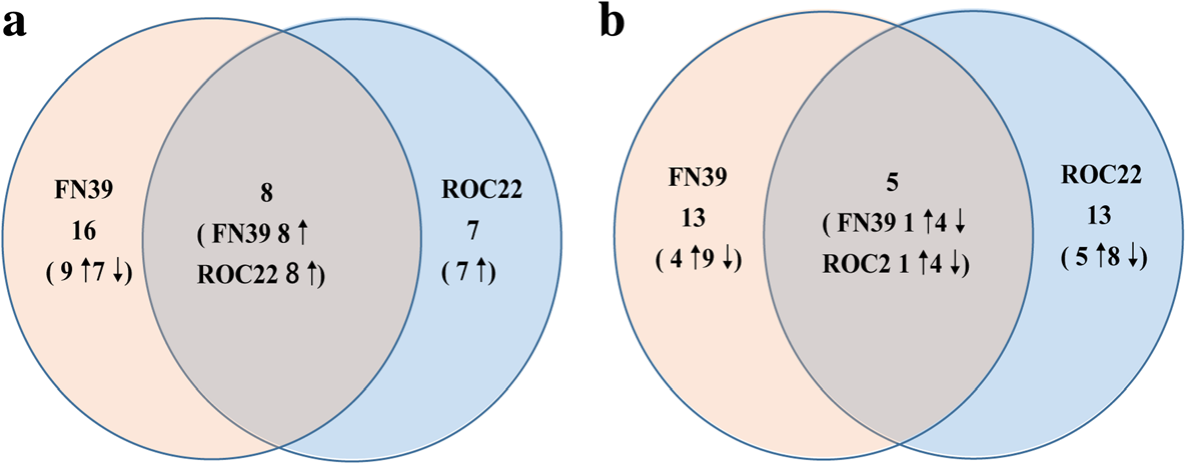
Distribution of differentially expressed miRNAs in FN39 and ROC22 cultivars. **a**) Known miRNAs; **b**) novel miRNAs

**Fig. 3:**
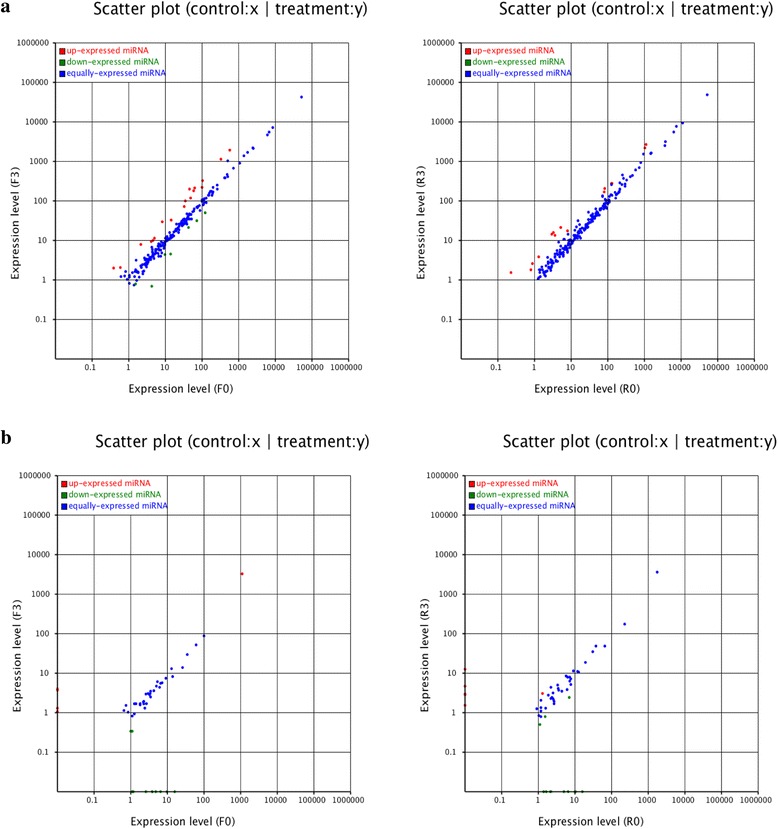
Scatter plots of differentially expressed miRNAs in 4 small RNA libraries. **a**) Known miRNAs; **b**) novel miRNAs. Green: significantly downregulated miRNAs; red: significantly upregulated miRNAs; blue: differentially expressed miRNAs (not significant). F0 and F3 represent the bud samples from sugarcane cultivar FN39 with 0 and 3 h cold (4 °C) treatment, respectively, while R0 and R3 represent the similar samples from cultivar ROC22 with 0 and 3 h cold (4 °C) treatment, respectively

To identify the known miRNAs, the clean reads were aligned with known miRNAs in miRBase. There were 219, 207, 210 and 209 known miRNAs identified in the F0, F3, R0 and R3 small RNA libraries, respectively (Additional file 3: Table S3). Compared with F0, F3 had 24 differentially expressed miRNAs (|log_2_ ratio| ≥ 1, *p* ≤ 0.05, Fig. 2a, Fig. 3a) including 17 upregulated and seven downregulated (Table 2). Similarly, 15 differentially expressed miRNAs (all upregulated) were identified upon comparison of R3 with R0 (Table 2). Among these differentially expressed miRNAs, there were eight common miRNAs (*miR2199*, *miR5054*, *miR5059*, *miR5072*, *miR5077*, *miR5152-3p*, *miR5813* and *miR8155*) in both cultivars; all of which were upregulated (Fig. 2a). Furthermore, some known differentially expressed miRNAs were identified in either FN39 or ROC22. Among them, 16 known miRNAs, including nine upregulated and seven downregulated miRNAs, were only found in FN39 libraries, accounting for 66.7% (Table 2), including *miR157a, miR160a*, *miR169b, miR6300*, *miR6196* and *miR64785*. Conversely, there were seven known differentially expressed miRNAs that existed only in ROC22, accounting for 46.7%, including *miR5272*, *miR863-5p*, *miR5242*, *miR5072*, *miR5221*, *miR395o-3p* and *miR1861d*.

**Table 2:** Known miRNAs with significantly differential expression in sugarcane buds following cold treatment

miRNA	sequence	F0-std	F3-std	R0-std	R3-std	Fold change	
						F0-F3	R0-R3
miR1310	GAGGCATCGGGGGCGCAACGCC	8.32	29.24	15.78	30.98	1.81**	0.97
miR1520d	ATCAGAACTGGTACGGACAA	574.08	1917.98	1131.16	2620.83	−1.74**	1.21
miR157a	TTGACAGAAGATAGAGAGCAC	4.65	9.68	4.34	4.26	1.06**	−0.02
miR2199	TGATAACTTGACGGATCGC	59.42	179.46	129.61	272.82	1.59**	1.07**
miR2916	GGGGCTCGAAGACGATCAGAT	46.30	198.06	128.65	255.78	−2.10**	0.99
miR5054	TCCCCACGGACGGCGCCA	50.00	117.65	81.84	164.77	1.23**	1.00**
miR5059	CGTTCCTGGGCAGCAACACCA	35.92	97.80	84.02	201.88	1.44**	1.26**
miR5072	GTTCCCCAGCGGAGTCGCCA	2.16	7.70	5.34	20.97	1.82**	1.97**
miR5077	TTCACGTCGGGTTCACCA	104.90	321.31	104.90	321.30	1.62**	1.62**
miR5152-3p	AGTCCTGCTATACCCACCA	0.59	2.02	1.30	3.76	1.77**	1.52**
miR5575	TGGATTTTGGAATGTTTTGGT	0.39	1.98	3.03	2.23	2.32**	−0.44
miR5671	CATGGTGGTGACGGGTGAC	32.73	70.96	72.74	126.88	1.12**	0.80
miR5813	ACAGCAGGACGGTGGTCATGGA	63.41	206.30	132.57	269.48	1.70**	1.02**
miR6196	GGGACGAGAAAGATGGGAGGA	5.12	11.12	17.93	21.15	1.12	1.09**
miR6300	GTCGTTGTAGTATAGTGGTG	101.38	217.53	216.10	284.36	1.10**	0.40
miR6478	CCGACCTTAGCTCAGTTGGTA	14.70	32.52	30.53	51.46	1.14**	0.75
miR8155	CGTAACCTGGCTCCGATACCA	4.14	9.20	8.10	16.96	1.15**	1.06**
miR894	GTTTCACGTCGGGTTCACCA	334.04	1108.12	1040.41	2131.64	1.72**	1.03
miR160a	TGCCTGGCTCCCTGTATGCCA	13.92	4.43	4.64	8.89	−1.65**	0.94
miR169b	CAGCCAAGGATGACTTGCCGG	43.54	20.76	38.28	39.38	−1.06**	0.04
miR169e-3p	GGCAGTCTCCTTGGCTAGC	73.19	31.02	14.24	21.84	−1.24**	0.62
miR397-5p	TTGACTGCAGCGTTGATGAGC	9.82	12.34	2.30	3.64	1.12**	0.66
miR528-5p	TGGAAGGGGCATGCAGAGGAG	123.62	49.19	303.86	262.15	−1.32**	−0.21
miR5532	ATGGAAATTGATGACAAAGGGG	1.58	0.77	1.46	1.20	−1.03 *	−0.28
miR1861d	TGCGTCATCAGGCAGGAACTGG	–	–	3.30	15.34	–	2.22**
miR395o-3p	TGAAGTGTTTGGGTGAACTC	2.60	1.64	0.88	2.56	−0.66	1.54**
miR5221	AACGAGATGGTGTTTTACTT	–	–	3.00	14.23	–	2.24**
miR5242	TATTTAGAACAGGCGATGTCA	1.26	1.49	3.68	13.24	0.24	1.84**
miR5272f	GAATTGATTTGTGTTGGATAAATT	3.27	2.64	0.80	1.78	−0.30	1.14**
miR394a	TTGGCATTCTGTCCACCTCC	4.26	7.78	0.88	0.74	1.03**	−0.96
miR863-5p	TTATAGTCTTGTGGATCAAAT	–	–	0.23	1.53	–	2.73**

F0 and F3 represent the bud sample from sugarcane cultivar FN39 with 0 h and 3 h cold (4 °C) treatment, respectively, while R0 and R3 represent the similar samples from cultivar ROC22, respectively. ** and * indicate a significant difference at p-value <0.01 and p-value <0.05, respectively. “---” indicates no expression

### Identification of novel miRNAs in sugarcane

A total of 151, 109, 140 and 132 novel miRNAs were discovered from the F0, F3, R0 and R3 libraries respectively using the Mireap software (Additional file 3: Table S3). There were 18 (five upregulated and 13 downregulated miRNAs) and 18 (six upregulated and 12 downregulated miRNAs) novel miRNAs expressed differentially in FN39 and ROC22 respectively after cold treatment for 3 h (|log_2_ ratio| ≥ 1, *p* ≤ 0.05, Fig. 2b, Fig. 3b, Table 3). Compared with known miRNAs, the novel miRNAs exhibited greater differential expression (a 10-fold up-regulation of *novel_mir_117* in R3 and 11-fold down-regulation of *novel_mir_72* in F 3). A proportion of novel miRNAs also showed cultivar-specificity, being expressed only in FN39 (13 novel, four upregulated and nine downregulated) or in ROC22 (13 novel, 5 upregulated and 8 downregulated).

**Table 3:** Novel miRNAs with significant differential expression in sugarcane buds following cold treatment

miRNA	sequence	F0-std	F3-std	R0-std	R3-std	Fold change	
						F3-F0	R3-R0
novel_mir_107	AGGGATGGTATGCACTGAAGC	0.01	3.71	0.01	4.55	8.54**	8.83**
novel_mir_117	AAGAGGAAGAGAGAGAGGGTG	0.01	0.01	0.01	12.33	–	10.27**
novel_mir_134	GAGGCAAGGAGGTGGAATAGAC	0.01	3.90	–	–	8.61**	–
novel_mir_136	AAGCAAGTTGGGGTAGGCTAA	0.01	1.30	–	–	7.02**	–
novel_mir_142	TGAAACGGTACGGCTGATAAGTT	0.01	1.06	–	–	6.73**	–
novel_mir_21	TAAATATATAACATCAGGACC	1.10	0.01	–	–	−6.79**	–
novel_mir_34	CGTACCACCGTCCGGGACTAA	1.14	0.01	–	–	−6.84**	–
novel_mir_37	TCCAATGCATACCGTCCCTAA	3.94	0.01	5.03	0.01	−8.62**	−8.97**
novel_mir_41	CCGAGAGGTAGGGCCGGTCGAA	6.55	0.01	12.83	10.34	−9.35**	–
novel_mir_54	TCCAAATTATAAGACGTTTTG	1.03	0.34	1.57	0.79	−1.60**	−1.00 *
novel_mir_57	GGGCTGGTTCGGCTGGTGGAA	1.10	0.01	–	–	−6.79**	–
novel_mir_68	ATAAGACGTTTTGGCTTTTCT	3.90	0.01	–	–	−8.61**	–
novel_mir_72	AAGAGGAAGAGAGAGAGGGTGA	16.09	0.01	16.17	0.01	−10.65**	−10.66**
novel_mir_74	TCTTGGATTTGCATTGGATGCC	1.18	0.01	–	–	−6.89**	–
novel_mir_79	CGGCCGACGCGCCGCGGCGGGC	2.64	0.01	2.19	0.01	−8.05**	−7.77**
novel_mir_80	TGGTTGTTGGCTGGCCATGGCTG	5.44	5.93	6.57	0.01	–	−9.36**
novel_mir_81	GCTAGAGGCAGCAACTGCATA	9.94	0.01	–	–	−9.96**	–
novel_mir_89	CAATCGTGGACCAACTAGGCT	4.85	0.01	3.53	4.01	−8.92**	0.18
novel_mir_91	CCTGCGTCGCACGGATTCGT	1114.45	3231.35	–	–	1.54**	–
novel_mir_97	GTCGTCGCCGTCGTCGTCGTC	1.10	0.34	1.19	0.79	−1.71**	−0.60
novel_mir_1	GGAATGTTGTCTGGTCGGAGA	9.31	7.37	10.48	0.01	−0.34	−10.03**
novel_mir_130	GGCATGGGAACATGTAGGAAGG	–	–	0.01	2.81	–	8.14**
novel_mir_171	TTTGAATAAGACGAGTGGTCA	–	–	10.33	0.01	–	−10.01**
novel_mir_183	TAAGCTGGCTGATGCTGTTTT	–	–	1.65	0.01	–	−7.37**
novel_mir_216	TCGAGGCCGAACGGATAAGTC	–	–	0.01	1.53	–	7.26**
novel_mir_228	TTTTTGGTGATTGATGACAAC	–	–	0.01	2.90	–	8.18**
novel_mir_28	AGGTCATGCTGTAGTTTCATC	–	–	1.30	3.02	–	1.21**
novel_mir_3	GGCTGAGCATGCCGCCGTCGAG	0.75	1.49	7.18	2.40	1.00	−1.58**
novel_mir_58	CGTCGCCGTCGTCGTCGTCGTC	2.29	1.64	1.11	0.50	−0.48	−1.16 *
novel_mir_64	AGGGGTTGTGCGGCGGTGCCC	–	–	1.42	0.01	–	−7.15**
novel_mir_67	AAAAACTTTGTACTAAAGCATA	–	–	2.27	0.01	–	−7.82**

F0 and F3 represent the bud sample from sugarcane cultivar FN39 with 0 h and 3 h cold treatment, respectively; R0 and R3 represent the bud sample from sugarcane cultivar ROC22 with 0 h and 3 h, respectively. ** and * indicate a significant differences at *p*-value <0.01 and *p*-value <0.05, respectively. “---” indicates no expression

### Functional analysis of target genes of differential expressed miRNAs

A total of 2805 target genes for 202 known expressed miRNAs were predicted using the Mireap software from the sugarcane EST database and the sugarcane unigene database (Additional file 4: Table S4). In order to provide an insight into their biological functions, GO and KEGG analyses of differentially expressed miRNAs were performed.

**Fig. 4:**
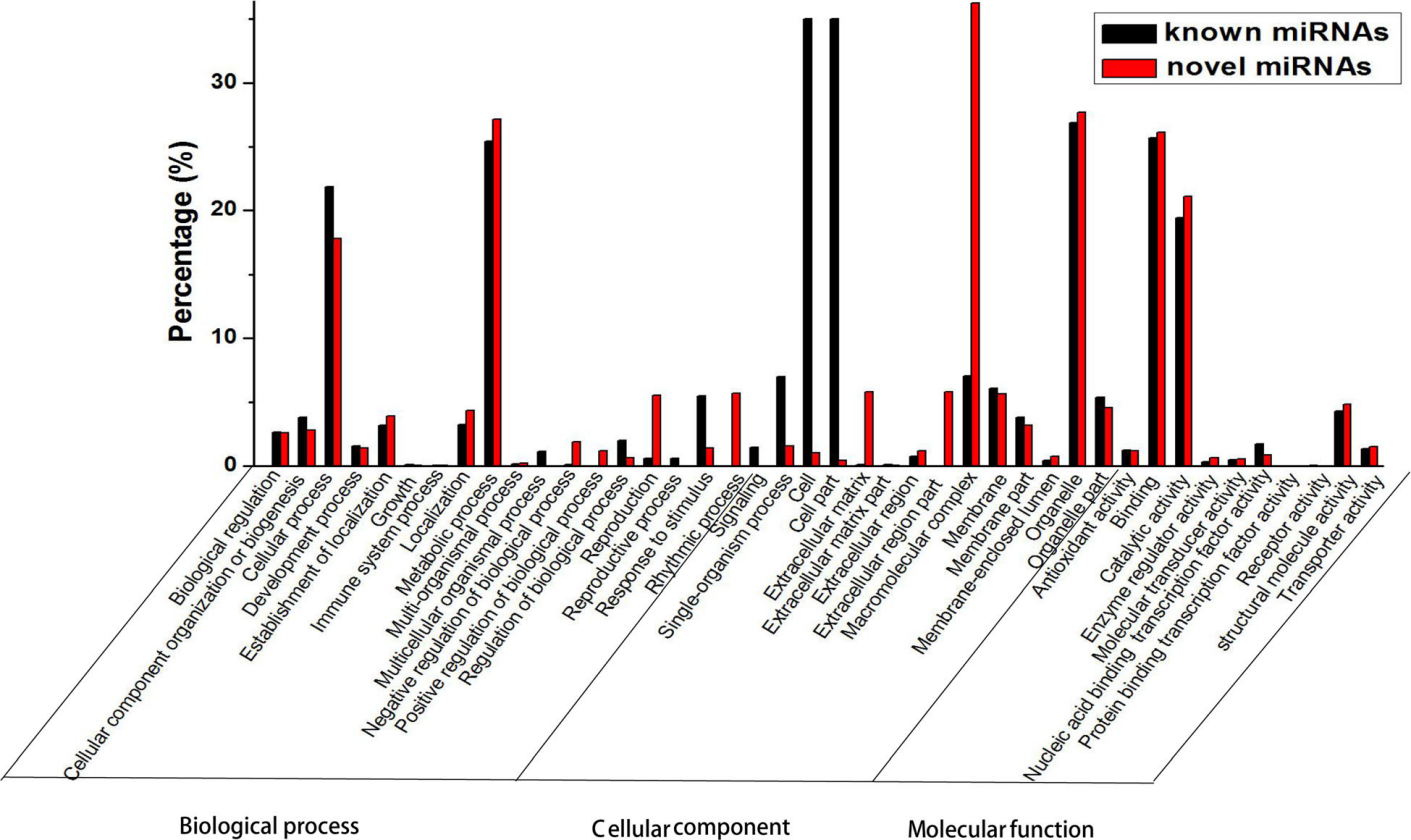
GO analysis of the predicted target genes for known and novel differentially expressed miRNAs

GO analysis of the predicted targets of differentially expressed miRNAs includes three ontologies: biological process, cellular component and molecular function (Additional file 5: Table S5). On the biological process, the result of the GO analysis indicated that the targets of the differentially expressed known and novel miRNAs were mostly involved in cellular and metabolic processes (Fig. 4). On the cellular component, the predicted targets of differentially expressed known miRNAs were mainly associated with cell and cell part, while for the novel miRNAs, their targets were mainly related with the macromolecular complex (Fig. 4). The molecular function of these targets was mainly related to the binding and catalytic activity (Fig. 4).

**Fig. 5:**
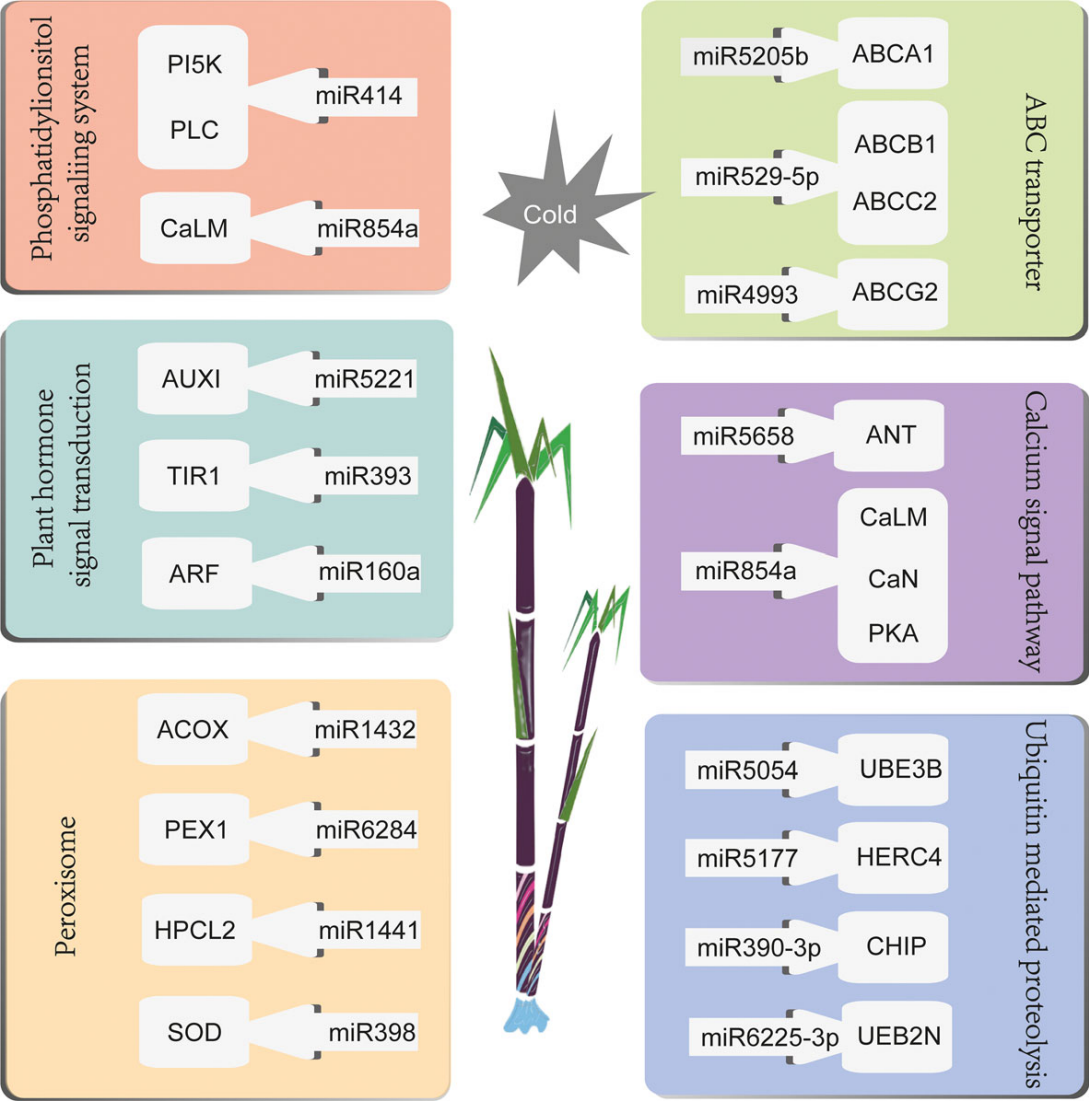
Predicted regulation networks between miRNAs and their target genes involved in six biological pathways in the sugarcane response to cold stress

**Fig. 6:**
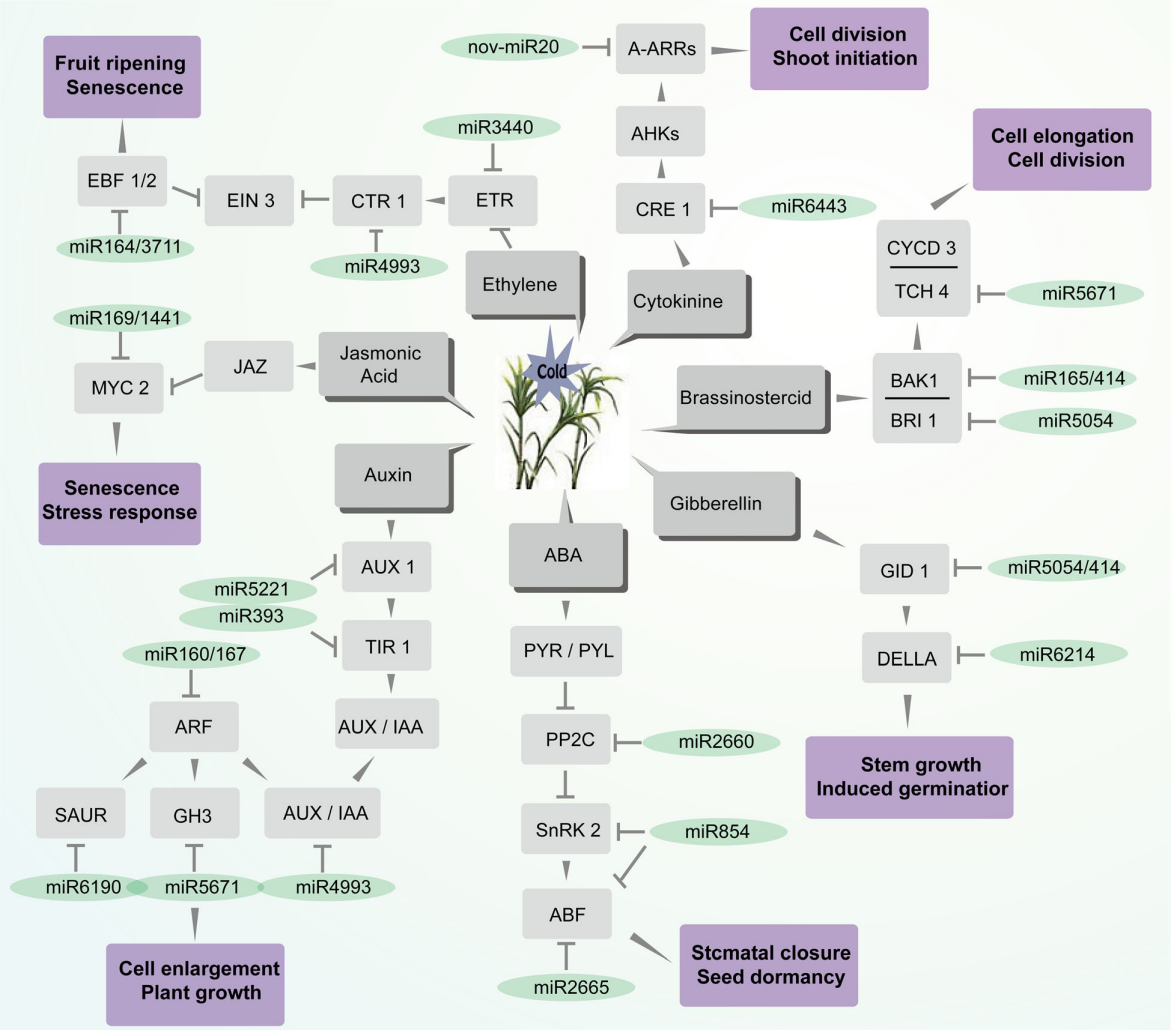
The involvement of miRNAs and their predicted targets into plant hormone signaling pathways in sugarcane response to cold stress. The *miR3440*-*ETR*, *miR4993*-*CTR1*, *miR164*/*3711*-*EBF1*/*2* were involved in the ethylene signal pathway which is related to the fruit ripening senescence. *miR6443*-*CRE*, nov-*miR20*-*A*-*ARRs* were involved in the cytokinine signal pathway which is related to the cell division shoot initiation. *miR5054*-*BRI1*, *miR165*/*414*-*BAK1*, *miR5671*-*TCH4* are involved in the brassinostercid signal pathway which is related to cell elongation and cell division. *miR5054*/*414*-*GID1*, *miR6214*-*DELLA* are involved in the gibberellin signal pathway which is related to stem *ABF* growth and induced germination. *miR2660*-*PP2C*, *miR854*-*SnRK2*/*ABF*, *miR2665*-*ABF* are involved in ABA signal pathway which is related to stomatal closure and seed dormancy. *miR5221*-*AUX1*, *miR393*-*TIR1*, *miR160*/*167*-*ARF*, *miR6190*-*SAUR*, *miR5671*-*GH3*, *miR4993*-*AUX*/*IAA* are involved in auxin signal pathway which is related to cell enlargement and plant growth. *miR169*/*1441*-*MYC2* are involved in jasmonic acid signal pathway which is related to senescence and stress response

KEGG analysis of target genes was performed (Additional files 6, 7, 8 and 9: Table S6-S9). In our study, six pathways related to cold stress were analyzed, including plant hormone signal transduction, ABC transporters, peroxisomes, phosphatidylinositol signaling systems, ubiquitin mediated proteolysis and calcium signal pathways (Fig. 5). There were a number of miRNAs that are associated with the regulation of hormone signal pathways (Fig. 6). These miRNAs could affect plants in a number of ways, including cell enlargement, stomatal closure, stem growth, germination, cell division, shoot initiation, fruit ripening and senescence through regulating their target genes.

### Expression analysis of miRNAs and their targets by RT-qPCR

**Fig. 7:**
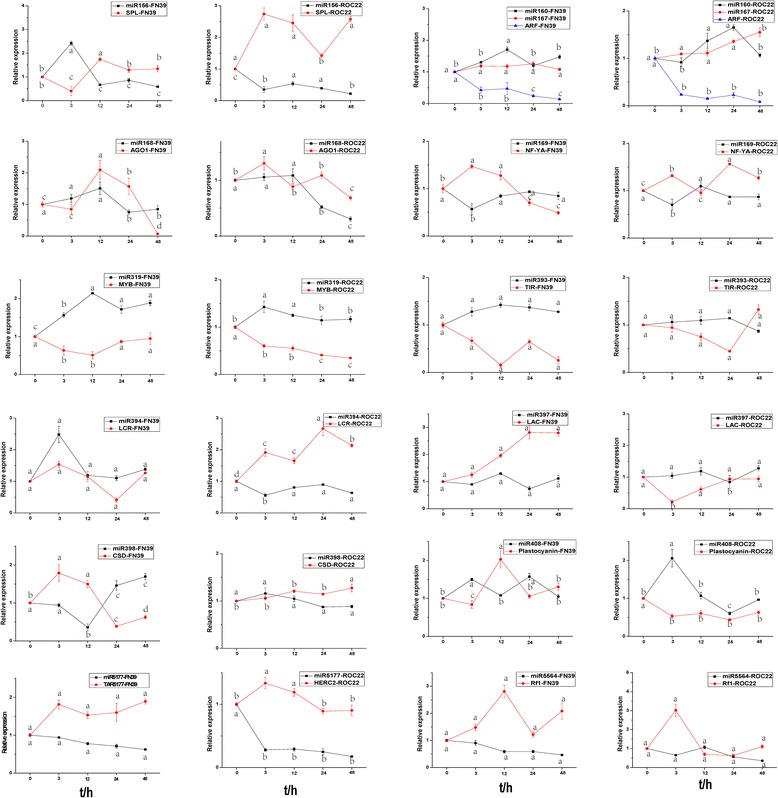
The expression profiles of 13 miRNAs and their targets in the two sugarcane cultivars FN39 and ROC22. The relative expression levels of miRNAs and their predicted target genes were normalized to the *18S rRNA* and *GAPDH* expression levels, respectively. Each bar represented as means of three replicates (*n* = 3) ± standard error. Lowercase letters in each graph attest significant differences within the same gene/miRNA over the treatment period, as determined by Duncan’s new multiple range test (*p*-value <0.05)

**Fig. 8:**
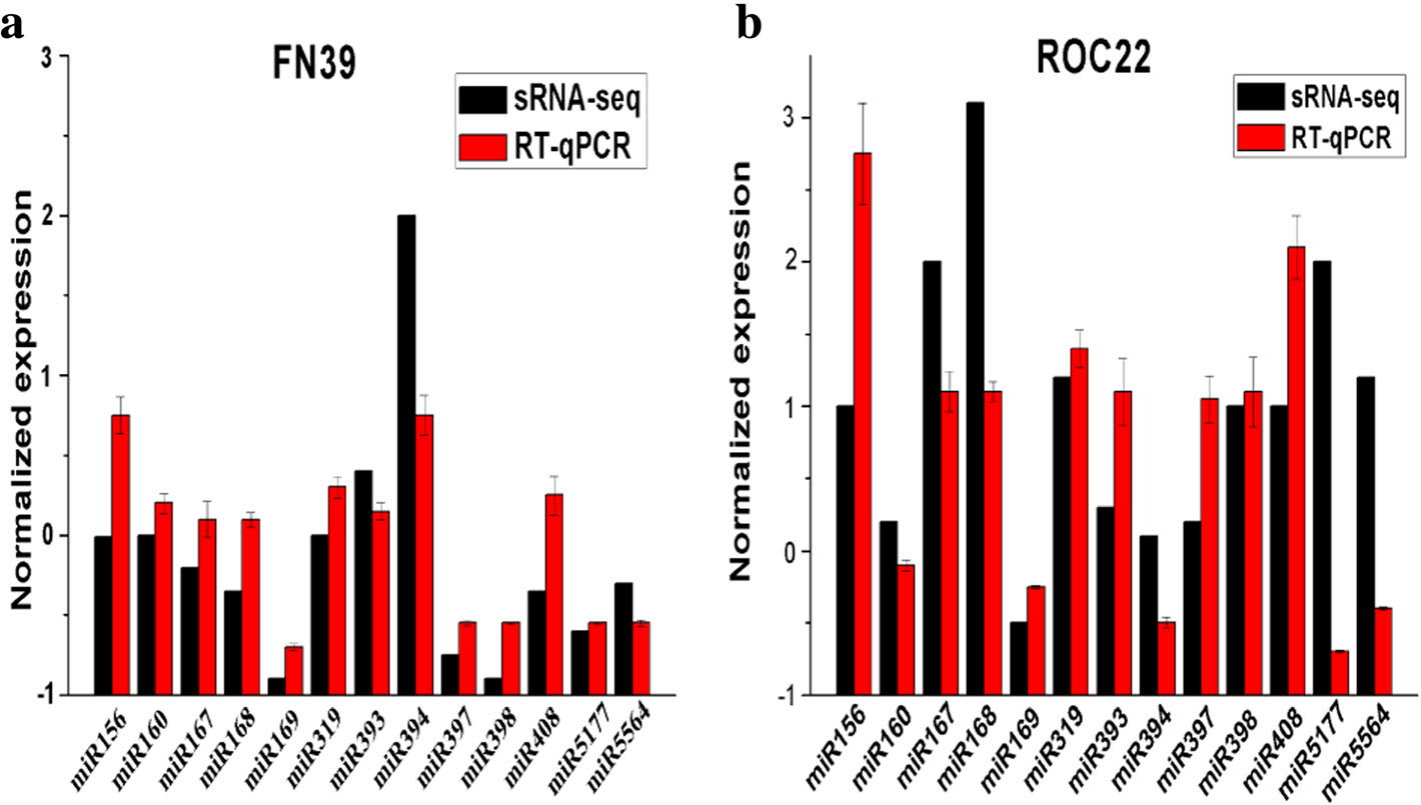
qRT-PCR validation of 13 randomly selected miRNAs identified by small RNA sequencing. **a**) miRNAs expressed in FN39. **b**) miRNAs expressed in ROC22. Sugarcane buds of FN39 and ROC22 treated with cold stress for 48 h were used as qRT-PCR samples. The data of qRT-PCR were normalized to the *18S rRNA* expression level and represented as means of three replicates (n = 3) ± standard error

RT-qPCR was used to detect the expression levels of miRNAs and their targets. A total of 13 miRNAs and 12 corresponding targets including four transcription factors and eight protein coding genes, were tested in the samples of the two sugarcane cultivars collected at different time points under cold stress (Fig. 7). In total, there were 11 miRNAs, sharing a similar expression profile with those in the deep sequencing data when detected by RT-qPCR. As shown in Fig. 8, the miRNA expression patterns (up- or down-regulation) as measured by quantitative analysis and the sequencing results of the miRNAs were largely consistent, except *miR394* and *miR5177*. From the expression profiles detected by RT-qPCR, there were several patterns among these 13 miRNAs. The transcripts of *miR160*, *miR167* and *miR319* were all induced by cold treatment in FN39 and ROC22. In contrast, the expressions of *miR169*, *miR5177* and *miR5564* were all suppressed. *MiR168* was induced at the early stage, but was inhibited afterwards. The other six miRNAs showed different expression patterns in FN39 and ROC22. *MiR156* showed a trend of increase firstly, then decrease in FN39, while it was downregulated in ROC22. The expression levels of *miR397* and *miR398* showed first decrease then increase in FN39, while the opposite trend was observed in ROC22. In FN39, the expression levels of *miR393*, *miR394* and *miR408* were all induced by the cold stress, while in ROC22, the expression levels of *miR393* and *miR408* were first increased then decreased, and the *miR394* was inhibited all the time. The different expression patterns of miRNAs in relative cold tolerance FN39 and relative cold sensitivity ROC22 may indicate their important roles in plant response to cold stress. Most of the above 13 miRNAs exhibited a negative correlation with their predicted targets in both cultivars (FN39 and ROC22) with the exception of *miR168* and *miR394* (Fig. 7)*.* The inaccuracy of target prediction or regulatory mechanism other than miRNAs modulating the expression of targets in sugarcane may explain this result.

### Transient expression analysis of miR156 in tobacco

**Fig. 9:**
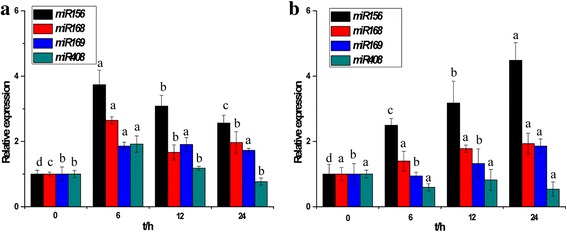
The expression of *miR156*, *miR168*, *miR169* and *miR408* in sugarcane under salt (**a**) and drought (**b**) stresses. The four miRNAs showed different responses to the salt and drought treatments. Compared with the other three miRNAs, *miR156* showed the largest differential expression. The other three miRNAs exhibited smaller differences compared to the control. *18S rRNA* was used as the internal control gene. Each bar represented as means of three replicates (n = 3) ± standard error. Lowercase letters in each graph attest significant differences within the same gene/miRNA over the treatment period, as determined by Duncan’s new multiple range test (p-value <0.05)

Homologues of *miR156*, *miR168*, *miR169* and *miR408* have been implicated in abiotic stresses [39, 40]. In addition to cold stress (Fig. 7), the expression profiles of these four miRNAs were detected under drought and salt stresses using stem loop RT-qPCR (Fig. 9). Compared with the other three miRNAs, *miR156* showed the largest differential expression, with 4-fold and 3-fold up-regulation respectively under drought and salt stresses. The other three miRNAs exhibited smaller differences compared to the control. This suggests a role for *miR156* in plant response to abiotic stress in sugarcane.

**Fig. 10:**
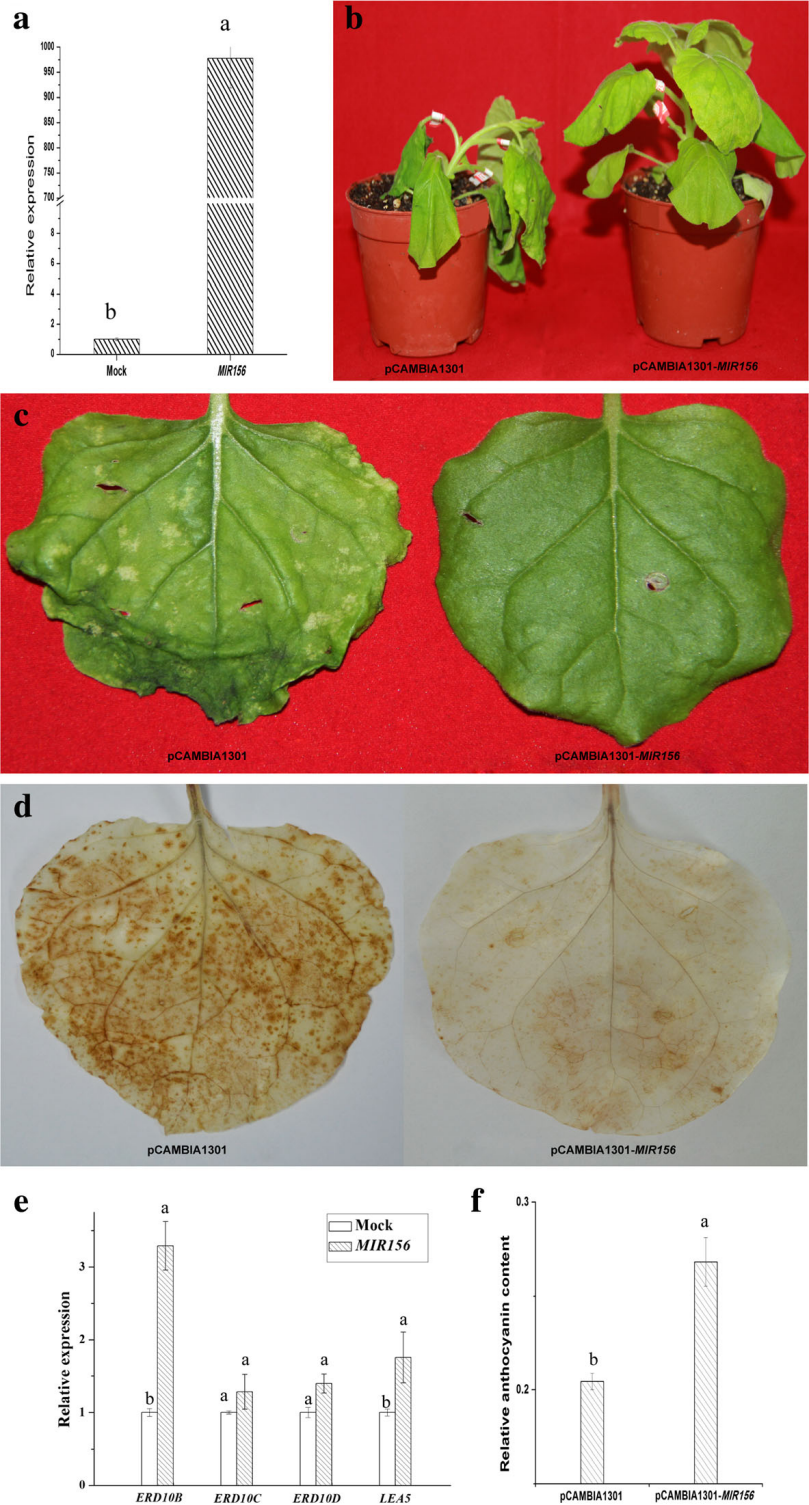
Functional analysis of *miR156* in tobacco infiltrated by *Agrobacterium tumefaciens* strain EHA105. **a**) The transcript analysis of *MIR156* in infiltrated tobacco leaves; **b**) and **c**) the phenotype observed in infiltrated tobacco leaves overexpressing pCAMBIA1301 and pCAMBIA1301-*MIR156* with 4 °C treatment for 24 h; **d**) DAB staining of tobacco leaves with 4 °C treatment for 24 h; **e**) expression analysis of cold response genes in infiltrated tobacco leaves with 4 °C treatment for 12 h. **f**) quantification of anthocyanin in tobacco leaves with 4 °C treatment for 24 h. A data point represents the mean ± SE (n = 3) (**a**, **e**, **f**). Lowercase letters in each graph attest significant differences within the same gene/miRNA over the treatment period, as determined by Duncan’s new multiple range test (p-value <0.05)

*MIR156* was transiently overexpressed in tobacco leaves using *Agrobacterium* expressing pCAMBIA1301-*MIR156*. Significant levels (*p*-value <0.05) of the *MIR156* transcript were detected (Fig. 10a), but not in the control. After 24 h cold treatment, the tobacco plants showed a visual phenotype. Compared with the control, the leaves overexpressing *MIR156* exhibited better growth status (Fig. 10b, c). A number of assays were performed on the tobacco leaves. The extent of H_2_O_2_ accumulation under cold stress was assayed using the DAB staining method. A deeper staining was produced in mock leaves, indicating lower H_2_O_2_ accumulation in leaves overexpressing *MIR156* (Fig. 10d). Three members of the early-responsive dehydration (*ERD*) gene family, *ERD10B*, *ERD10C* and *ERD10D*, which are downstream genes in the ICE-CBF-COR pathway [41], and the *LEA* gene, whose encoded protein protects cells from water stress [42], were upregulated in pCAMBIA1301-*MIR156* overexpressing tobacco leaves after 12 h cold treatment (Fig. 10e). Additionally, under cold stress, anthocyanin levels in leaves overexpressing pCAMBIA1301-*MIR156* were higher than those in the control (Fig. 10f). These results suggest that tobacco leaves overexpressing *MIR156* have enhanced cold tolerance. In addition, the transient expression of the other three miRNAs, *miR168*, *miR169* and *miR408*, produced no obvious phenotype or difference in reactive oxygen species (ROS).

## Discussion

### Small RNA analysis of sugarcane response to cold stress

Cold stress is a major limiting factor in the distribution, yield and quality of crops [11]. Although the cold signaling pathway has been well studied [11, 14], the role of small RNAs in low temperature sensing in plants remains unclear. In recent years, several researchers have found a critical role of miRNA in abiotic stress [43, 44]. Cold-related miRNAs in plants have been identified using various methods. Zhou et al. (2008) [16] found 19 upregulated miRNA genes of 11 miRNA families in *Arabidopsis thaliana* under cold stress using a computational, transcriptome-based approach. In *Brachypodium distachyon,* 27 conserved and 129 predicted miRNAs involved in the cold stress response were identified by high-throughput sequencing and whole-genome-wide data mining [17]. In *Poncirus trifoliate* (L.) *Raf*., a total of 107 conserved and five potentially novel miRNAs were characterized before and after cold treatment through deep sequencing [45]. In this study, a total of 412 sugarcane miRNAs, including 261 known and 151 predicted novel miRNAs, were discovered in two cultivars exhibiting differences in cold tolerance by small RNA deep sequencing and bioinformatics analysis, enriching the present miRNA resources in sugarcane. This is far more than previously identified in other species, except for the possibility of the parameters used were different, it is most probably related to the larger genome and complex genetic background of sugarcane.

These small RNAs obtained in this study were divided into several classes. Due to the limited knowledge of sugarcane genome information, the unannotated small RNAs occupied the majority, which was consistent with the halophyte *Halostachys caspica* [30] and *Morus alba* L. [46]. According to a previous report, the 21 nt and 24 nt length sRNA sequences are generated by different DCL proteins [6]. Small RNAs with 20–22 nucleotides are generated by DCL1. Longer small RNAs, 23–25 nucleotides in length, are processed by DCL3. The DCL2 and DCL4 generate the 22 and 24 nucleotides respectively [47]. Usually, the length of miRNAs corresponds to 21 nt or 22 nt, while the siRNAs are distributed in the 24 nt [6]. The 24 nt and 21 nt small RNA length distribution in sugarcane was consistent with the phenomenon in *Panicum virgatum* [48], *Fragaria ananassa* [49], rice [50] and *H. caspica* [30], which verified our data.

Among the total 412 identified miRNAs, 62 miRNAs exhibited significant expression differences (|log_2_ ratio| ≥ 1, *p* ≤ 0.05) between the cold-treated and the control samples. Differentially expressed miRNAs were further validated by RT-qPCR. According to previous reports, *miR160/167* and *miR393* are involved in the auxin response by targeting of auxin response factors (*ARF*) and transport inhibitor response (*TIR*) genes, respectively [51, 52]. In this study, *miR167* and *miR393* were upregulated in both cultivars, while *miR160* was upregulated in FN39 (relative cold tolerance cultivar) and downregulated in ROC22 (relative cold sensitivity cultivar). In *Lycopersicon esculentum* Mill studies using stem-loop RT-qPCR, *miR167* and *miR393* increased at 4 °C at early time points (0, 1, 4 and 16 h) [53]. In 2-week-old *Arabidopsis* seedlings, *miR393* was strongly induced by cold, NaCl, dehydration and ABA treatments [54]. The up-regulation of *miR167* was found in *Triticum aestivum*, suggesting that *miR167* and the trans-acting short-interfering RNA-auxin response factor (tasiRNA-ARF) have a role in regulating the auxin signaling pathway and the developmental response to cold stress [55]. Under unstressed conditions, low levels of these miRNAs may be sufficient for the fine-tuning of their targets. Conversely, under stress conditions, upregulated miRNAs can reduce ARF and TIR transcript levels (Fig. 7), suppressing ARF-mediated auxin responsive gene expression, leading to plant growth and development attenuation and eventually enhancing plant stress tolerance.

There was up-regulation of *miR168* and *miR408* under cold stress in both the cold tolerant and less cold tolerant sugarcane cultivars. Liu et al. (2008) [39] identified 14 stress-inducible miRNAs using microarray data in *Arabidopsis*, and found *miR168* to be responsive to cold stress. The increased expression of *miR408* could lead to the improved tolerance of *Arabidopsis* to cold and oxidative stresses and the enhancement of cellular antioxidant capacity, manifested by the lower level of ROS and induction of genes related to anti-oxidative function [56]. *MiR319* showed upregulated expression in both cultivars in this study, which was consistent with previous research which identified its positive role in the response of sugarcane to cold stress [18]. Some miRNAs exhibited differential expression in the two cultivars: *miR156*, *miR160* and *miR394* were upregulated in the cold tolerant FN39, but downregulated in the cold sensitive ROC22, while *miR397* and *miR398* were downregulated in FN39 and upregulated in ROC22. The opposite expression changes of these miRNAs in these cultivars may be a key factor leading to difference in cold tolerance. *MiR156* is a conserved miRNA in many plant species and its function in plant growth and development has been studied during events such as lateral root development [57] and floral transition [58]. *MiR156* also has a role in plant response to abiotic stress, such as drought and salt stresses [59].

As the function of miRNAs is performed by targeting of mRNAs, the target genes were predicted with certain described criteria. Several targets of conserved miRNAs in this study, such as the SQUAMOSA promoter-binding protein-like gene (*SPL*, target of *miR156*) and nuclear factor Y gene (*NF-Y*, target of *miR169*), have been reported in some other plant species, indicating the miRNAs and its targets may have a similar role in different plant species [60]. In our study, a total of 4002 targets were predicted for the 259 miRNAs, showing that some miRNAs have more than one target, which is consistent with earlier reports in *Glycine max* seeds [61] and trifoliate orange [45]. Another reason for the large amount of predicted targets may be the lack of sufficient sugarcane genome sequences and annotation information, leading to inaccuracies in prediction. Among the targets, transcription factors were included and involved in the response of sugarcane to cold stress, such as the *ARF*, *TIR*, *MYB* and *NF*-*Y*. Transcription factors control a series of downstream genes by binding *cis*-elements in promoter regions [62]. It is conceivable that miRNAs regulating the transcription factors will exert a more extensive influence on gene expression. However, the function of miRNAs and their targets needs to be validated through further experiments.

### Functional analysis of *miR156* in tobacco under abiotic stress

Several miRNAs, including *miR156*, *miR168*, *miR169* and *miR408,* which have been studied in plant response to abiotic stresses [39, 40], were selected for further functional investigation. The highly conserved *miR156* family has been reported as a key factor in the regulation of plant growth and development by targeting the *SPL* genes. In *Gossypium hirsutum* L., the *miR156* was upregulated under 0.25% NaCl or 2.5% PEG stresses in roots [63]. In *Zea mays* L., *miR156* showed a modest up-regulation in two inbred lines under both salt and drought stresses, but also a strong higher expression in the hybrid lines based on northern blot detection. *SPL*, a target of *miR156*, showed opposite expression patterns under abiotic stresses, indicating the role of miR156 as a negative regulator in maize during this process [64]. A similar positive role of *miR156* in sugarcane was shown in our RT-qPCR results, with a 3.7-fold and 4.5-fold up-regulation under salt and drought stresses, respectively.

The role of *miR156* in cold stress has been shown in rice [20]. Transgenic rice with overexpressed *OsmiR156k* exhibited a lower survival rate, proline content, chlorophyll II, and down-regulation of cold-sensitive genes under cold stress, indicating decreased cold tolerance. They concluded that *miR156k* worked as a negative regulator of plant tolerance to cold stress [20]. In our work, *miR156* showed a positive role in the response of tobacco to cold stress, supported by better growth status and lower ROS accumulation. Additionally, 4 cold-related genes (*ERD10B*, *ERD10C*, *ERD10D* and *LEA* genes) all showed higher expression levels in tobacco leaves when *MIR156* was overexpressed than those in the control. This may be due to the fact that *miR156* exerts various affects in different plant species.

Anthocyanin level has been shown to be responsive to many stresses including cold, salt, UV-B and biotic stresses, and protect plants from damage by scavenging the free radicals [65, 66]. Gou et al. (2011) [38] concluded that increased *miR156* could promote the accumulation of anthocyanins, while reduced *miR156* would be conducive to flavonol accumulation in *Arabidopsis*. During this process, *SPL9*, one of the *miR156* targets, inhibits the expression of anthocyanin synthesis genes by interacting with the production of the anthocyanin pigments1 (PAP1) component of the MYB-BHLH-WD40 complex, resulting in the interference with the regulation of anthocyanin accumulation [38]. Due to the fact that the greater accumulation of anthocyanins in *miR156* transiently overexpressed tobacco leaves was observed, it is possible that the overexpression of sugarcane *miR156* leads to the accumulation of anthocyanins to prevent the over production and accumulation of ROS in tobacco leaves. This deduction was consistent with the observation of growth status and DAB staining, in which the *MIR156* overexpressing tobaccos displayed better growth status and less ROS accumulation. Considering the better growth status, lower ROS level, higher anthocyanins level with the higher expression levels of four cold-related genes in *MIR156* overexpressing tobaccos than those in control, it indicated that the *miR156* plays a positive role in plant response to cold stress.

## Conclusions

In this study, small RNA sequencing of sugarcane with cold treatment and comprehensive analysis of the cold-responsive miRNAs and their targets were performed. The results showed that miRNAs played an important role in sugarcane under cold stress by regulating several biological processes, including plant hormone signal transduction, ABC transporter function, peroxisomes, phosphatidylinositol signaling systems, ubiquitin mediated proteolysis and calcium signaling pathways. In addition, the role of *miR156* in plant response to cold stress was also studied. These findings provide the valuable information for further functional characterization of miRNAs in sugarcane under cold stress.

## Additional files

Additional file 1: Table S1. The stem loop primers of miRNAs in sugarcane (DOCX 14 kb)

Additional file 2: Table S2. The real time PCR primers of miRNAs and target gene in sugarcane (DOCX 15 kb)

Additional file 3: Table S3. The known and novel miRNAs in 4 small RNA libraries (F0, F3, R0 and R3) (XLSX 30 kb)

Additional file 4: Table S4. Prediction of target genes of the known and novel miRNAs (XLSX 208 kb)

Additional file 5: Table S5. GO analysis of the predicted targets of known and novel miRNAs (XLSX 243 kb)

Additional file 6: Table S6. KEGG analysis of the predicted targets of known miRNAs in F3/F0 (XLSX 12 kb)

Additional file 7: Table S7. KEGG analysis of the predicted targets of known miRNAs in R3/R0 (XLSX 11 kb)

Additional file 8: Table S8. KEGG analysis of the predicted targets of novel miRNAs in F3/F0 (XLSX 15 kb)

Additional file 9: Table S9. KEGG analysis of the predicted targets of novel miRNAs in R3/R0 (XLSX 12 kb)

Additional file 10: Figure S1. The stem longitudinal section of sugarcane FN39 (relative cold tolerance) and ROC22 (relative cold sensitivity) in plant tip part after cold stress, indicating that more serious damage was observed in ROC22 in its stem tissues and growing point (TIFF 31806 kb)

Additional file 11: Figure S2. Validation of the cold-stress treatment in sugarcane. The expression patterns of three cold responsive genes, *CBF1*, *CBF3* and *NAC23*, were detected by RT-qPCR in sugarcane FN39 (A) and ROC22 (B) cultivars. The relative expression at 0 h is equal to 1. *GAPDH* was used as internal control. Error bars show the range of duplicate analysis of sample in RT-qPCR. Lower case letters in each graph attest significant differences within the same gene/miRNA over the treatment period, as determined by Duncan’s new multiple range test (*p*-value <0.05) (TIFF 2258 kb)

## Abbreviations

*18S* rRNA: 18S ribosomal RNA
*ARF*: auxin response factor gene
*CBF*: C-repeat binding factor gene
DAB: 3, 3′-diaminobenzidine
F0: samples of FN39 cultivar without cold treatment
F3: samples of FN39 cultivar with 3 h cold treatment
*GAMyB*: *Myb* transcription factor
*GAPDH*: glyceraldehyde-3-phosphate dehydrogenase
*ICE1*: inducer of C-repeat binding factor expression 1 gene
KEGG: Kyoto Encyclopedia of Genes and Genomes
*NF-Y*: nuclear factor Y gene
PAP1: production of the anthocyanin pigments1
*PCF5*: *TCP* transcription factor gene
R0: samples of ROC22 cultivar without cold treatment
R3: samples of ROC22 cultivar with 3 h cold treatment
RISC: RNA-induced silencing complex
ROS: reactive oxygen species
rRNA: ribosomal RNA
RT-qPCR: reverse transcription quantitative real-time polymerase chain reaction
scRNA: small cytoplasmic RNA
snoRNA: small nucleolar RNA
snRNA: small nuclear RNA
*SPL*: SQUAMOSA promoter-binding protein-like gene
tasiRNA-ARF: trans-acting short-interfering RNA-auxin response factor
*TIR*: transport inhibitor response gene
tRNA: transfer RNA
