# Supplementary material for: miRNA alteration is an important mechanism in sugarcane response to low-temperature environment
Source: BMC Genomics. 2017 Oct 30;18:833. doi: 10.1186/s12864-017-4231-3 (PMC5661916; doi:10.1186/s12864-017-4231-3)
Supplement: Supplementary file 1 — The stem loop primers of miRNAs in sugarcane (DOCX 14 kb) [file 12864_2017_4231_MOESM1_ESM.docx]

**Table S1. The stem loop primers of miRNAs in sugarcane**

| **miRNAs** | **sequence** | **Stem loop primers** |
| --- | --- | --- |
| miR156 | TGACAGAAGAGAGTGAGCAC | CTCAACTGGTGTCGTGGAGTCGGCAATTCAGTTGAGGTGCTCAC |
| miR160 | GCGTGCAAGGAGCCAAGCATG | CTCAACTGGTGTCGTGGAGTCGGCAATTCAGTTGAGCATGCTTG |
| miR167 | TGAAGCTGCCAGCATGATCTGA | CTCAACTGGTGTCGTGGAGTCGGCAATTCAGTTGAGTCAGATCA |
| miR168 | CCCGCCTTGCACCAAGTGAAT | CTCAACTGGTGTCGTGGAGTCGGCAATTCAGTTGAGATTCACTT |
| miR169 | GGCAGTCTCCTTGGCTAGC | CTCAACTGGTGTCGTGGAGTCGGCAATTCAGTTGAGGCTAGCCA |
| miR319 | AGTGGATGGCGCGGGAGCTAA | CTCAACTGGTGTCGTGGAGTCGGCAATTCAGTTGAGTTAGCTCC |
| miR393 | CTCCAAAGGGATCGCATTGAT | CTCAACTGGTGTCGTGGAGTCGGCAATTCAGTTGAGATCAATGC |
| miR394 | TTGGCATTCTGTCCACCTCC | CTCAACTGGTGTCGTGGAGTCGGCAATTCAGTTGAGGGAGGTGG |
| miR397 | TTGACTGCAGCGTTGATGAGC | CTCAACTGGTGTCGTGGAGTCGGCAATTCAGTTGAGGCTCATCA |
| miR398 | GCAGGTGATGAGAACAAGA | CTCAACTGGTGTCGTGGAGTCGGCAATTCAGTTGAGTCTTGTTC |
| miR408 | ACAGGGATGAGGCAGAGCATG | CTCAACTGGTGTCGTGGAGTCGGCAATTCAGTTGAGCATGCTCT |
| miR5177 | TAGGGTGTAAAACAGACGGTT | CTCAACTGGTGTCGTGGAGTCGGCAATTCAGTTGAGAACCGTCT |
| miR5564 | TGGGGAAGCAATTCGTCGAACA | CTCAACTGGTGTCGTGGAGTCGGCAATTCAGTTGAGTGTTCGAC |
