# Supplementary material for: miRNA alteration is an important mechanism in sugarcane response to low-temperature environment
Source: BMC Genomics. 2017 Oct 30;18:833. doi: 10.1186/s12864-017-4231-3 (PMC5661916; doi:10.1186/s12864-017-4231-3)
Supplement: Supplementary file 2 — The real time PCR primers of miRNAs and target gene in sugarcane (DOCX 15 kb) [file 12864_2017_4231_MOESM2_ESM.docx]

**Table S2. The real time PCR primers of miRNAs and genes in sugarcane**

| **Genes** | **Primer-Forward** | **Primer-Reverse** | **Accession number** | **Reference** |
| --- | --- | --- | --- | --- |
| *miR156* | ACACTCCAGCTGGGTGACAGAAGAGAGT | TGGTGTCGTGGAGTCG | - | - |
| *miR160* | ACACTCCAGCTGGGGCGTGCAAGGAGCCA | TGGTGTCGTGGAGTCG | - | - |
| *miR167* | ACACTCCAGCTGGGTGAAGCTGCCAGCATG | TGGTGTCGTGGAGTCG | - | - |
| *miR168* | ACACTCCAGCTGGGCCCGCCTTGCACCAA | TGGTGTCGTGGAGTCG | - | - |
| *miR169* | ACACTCCAGCTGGGGGCAGTCTCCTTG | TGGTGTCGTGGAGTCG | - | - |
| *miR319* | ACACTCCAGCTGGGAGTGGATGGCGCGGG | TGGTGTCGTGGAGTCG | - | - |
| *miR393* | ACACTCCAGCTGGGCTCCAAAGGGATCGC | TGGTGTCGTGGAGTCG | - | - |
| *miR394* | ACACTCCAGCTGGGTTGGCATTCTGTCC | TGGTGTCGTGGAGTCG | - | - |
| *miR397* | ACACTCCAGCTGGGTTGACTGCAGCGTTG | TGGTGTCGTGGAGTCG | - | - |
| *miR398* | ACACTCCAGCTGGGGCAGGTGATGAGA | TGGTGTCGTGGAGTCG | - | - |
| *miR408* | ACACTCCAGCTGGGACAGGGATGAGGCAG | TGGTGTCGTGGAGTCG | - | - |
| *miR5177* | ACACTCCAGCTGGGTAGGGTGTAAAACAG | TGGTGTCGTGGAGTCG | - | - |
| *miR5564* | ACACTCCAGCTGGGTGGGGAAGCAATTCGT | TGGTGTCGTGGAGTCG | - | - |
| *SPL* | TTCCGCTGTACCCTCCAATGTT | GCTGTGCCTGTGGTCTCGCTT | XM_021459861.1 | *Sorghum bicolor* |
| *ARF* | CATCACCACCACCATTTCCTTC | GCATGCCTGGCTCCCTGTAT | NM_001165660.1 | *Zea mays* |
| *AGO1* | GAGAAGGGACAAGTAAGCG | TAAAATAATAATTCCGGGG | XM_015770610.1 | *Oryza sativa* |
| *NF-YA* | CCGCTGAAGAGCCCATTTATGT | AGATGCCGAGACTCGTGGAGGT | NM_001176424 | *Zea mays* |
| *MYB* | GGACCATTTGTGGGCCTAGA | TGGCAGCCATGCCTTTTTA | XM_015766849.1 | *Oryza sativa* |
| *TIR* | GCTGATGCTGTAACAGGGCAGC | GGAACACATTATCCGTGAGGCG | XM_021461469 | *Sorghum bicolor* |
| *LCR* | TGAAGGCGAAGCGTATGAACC | CATGTCAGGCCAAGGCGCAG | NM_102496.4 | *Arabidopsis thaliana* |
| *LAC* | GACGAGACGACACGAGGAA | CCCAGCACAAGCAAAAGAT | XM_014770382.1 | *Glycine max* |
| *CSD* | AAACAAAGCAGGCACAAAGTAG | GCAATAGCAGTGAAAGCAATAG | AFO59568.1 | *Saccharum* spp. |
| *Plastocyanin* | TTTTGGTTTGCTGGAGGAA | ATCAGGAGGGACGCGTTTT | XM_015788217.1 | *Oryza sativa* |
| *HERC2* | GTGAGGGTCATTGCTGTAGGTGC | CGGATGTGATCTTGGTTGGGTAT | KT832066.1 | *Helianthus annuus* |
| *Rf1* | GGCCTGTCCTTCGACAAATGG | TAAATACCCTCACTGCCTCCC | XM_015758797.1 | *Oryza sativa* |
| *18S rRNA* | CTACGTCCCTGCCCTTTGTACA | ACACTTCACCGGACCATTCAA | SCFRRE06 | *Oryza sativa* |
| *CBF1* | CGGACGGTTCATTTCATTCTC | GCTTCTTCTTGAACTCCTCCC | NM_001158421.1 | *Zea mays* |
| *CBF3* | GCGGAAGGGTTGCTCGTGGA | GGGGGCTTTGGGGTGGTTGT | XM_015755426.1 | *Oryza sativa* |
| *NAC23* | GAGAAGACCAACTGGATCA | GCCCTCCCTTCTTGTTGTAG | AY742218 | *Saccharum* spp. |
| *GAPDH* | CACGGCCACTGGAAGCA | TCCTCAGGGTTCCTGATGCC | CA254672 | *Saccharum* spp. |
