# Supplementary figures and images for: miRNA alteration is an important mechanism in sugarcane response to low-temperature environment
Source: BMC Genomics. 2017 Oct 30;18:833. doi: 10.1186/s12864-017-4231-3 (PMC5661916; doi:10.1186/s12864-017-4231-3)

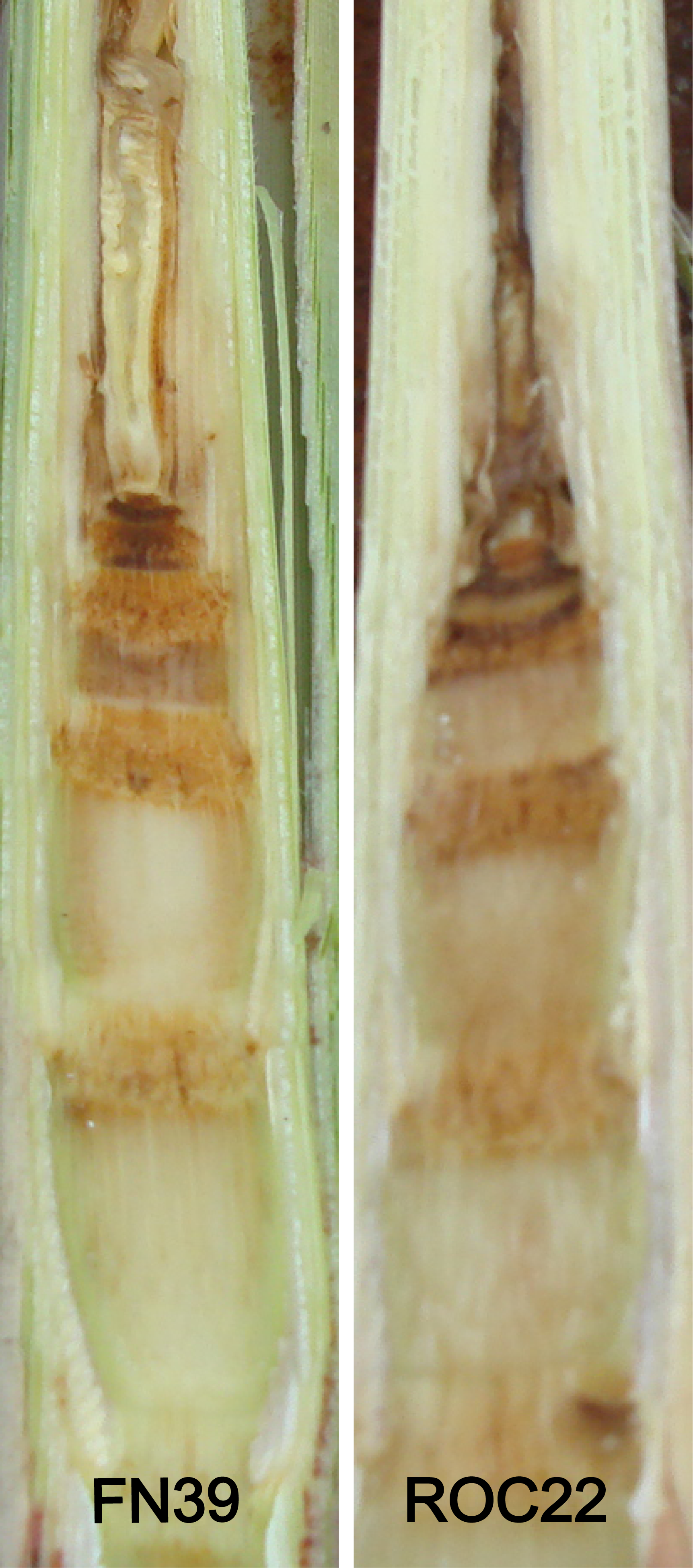

Supplement: Supplementary file 10 — The stem longitudinal section of sugarcane FN39 (relative cold tolerance) and ROC22 (relative cold sensitivity) in plant tip part after cold stress, indicating that more serious damage was observed in ROC22 in its stem tissues and growing point (TIFF 31806 kb) [file 12864_2017_4231_MOESM10_ESM.tif]

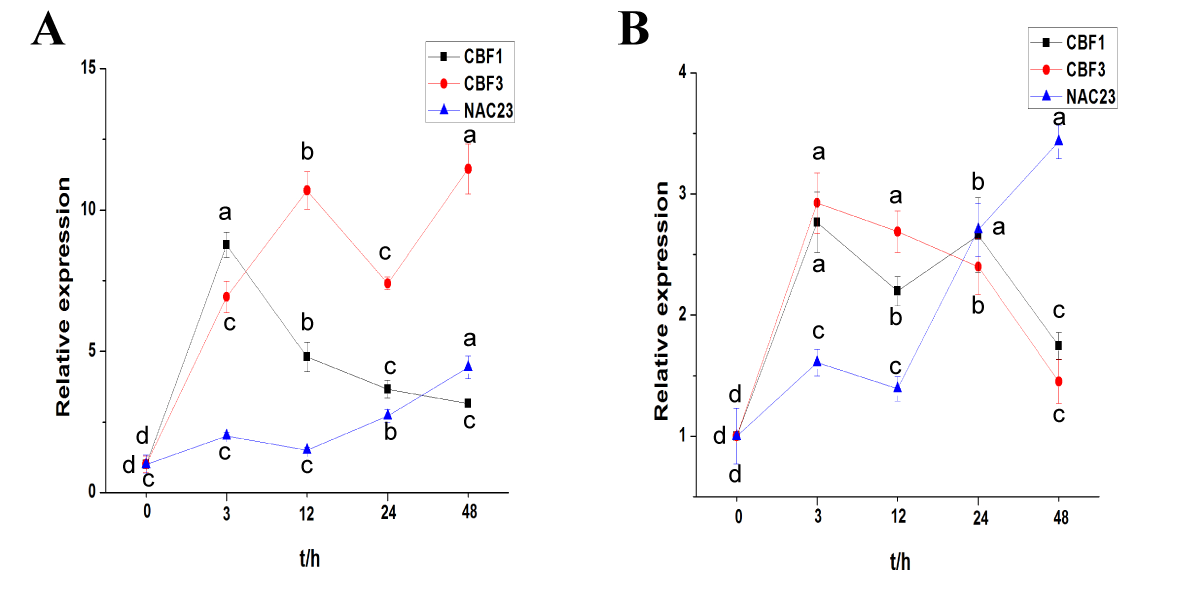

Supplement: Supplementary file 11 — Validation of the cold-stress treatment in sugarcane. The expression patterns of three cold responsive genes, CBF1, CBF3 and NAC23, were detected by RT-qPCR in sugarcane FN39 (A) and ROC22 (B) cultivars. The relative expression at 0 h is equal to 1. GAPDH was used as internal control. Error bars show the range of duplicate analysis of sample in RT-qPCR. Lower case letters in each graph attest significant differences within the same gene/miRNA over the treatment period, as determined by Duncan’s new multiple range test (p-value <0.05) (TIFF 2258 kb) [file 12864_2017_4231_MOESM11_ESM.tif]
